# Diagnostic Uncertainty in a Patient With Late-Onset Hypogonadism-Like Symptoms: A Case Report

**DOI:** 10.7759/cureus.103830

**Published:** 2026-02-18

**Authors:** Kenta Ichino, Hisamitsu Ide, Nobuo Okui, Shigeo Horie

**Affiliations:** 1 Data Science and Informatics for Genetic Disorders, Graduate School of Medicine, Juntendo University, Tokyo, JPN; 2 Urology, Graduate School of Medicine, Juntendo University, Tokyo, JPN; 3 Innovative Longevity, Graduate School of Medicine, Juntendo University, Tokyo, JPN; 4 Urology, Yokosuka Urogynecology and Urology Clinic, Kanagawa, JPN; 5 Mathematics, Kanagawa Dental University, Kanagawa, JPN

**Keywords:** clinical overlap, diagnosis delay, diagnostic reasoning, diagnostic thresholds, late-onset hypogonadism, mental assessment, nonspecific findings, response to treatment, testosterone replacement therapy (trt), undiagnosed case

## Abstract

Late-onset hypogonadism (LOH) is a clinical concept that integrates symptoms with decreased testosterone and affects sexual function, physical vitality, and psychological or behavioral domains. In patients whose complaints traverse multiple specialties, symptom nonspecificity can constrain hypothesis updating. Variation in biochemical assessment, including measurement timing and thresholds, can amplify reliance on treatment response.

A 52-year-old Japanese man presented with fatigue, awakening after sleep onset, flushing, palpitations, and erectile dysfunction despite internal medicine evaluations. Vital signs, electrocardiography, blood tests, CT, and MRI suggested no organic disease, and he was referred to urology for suspected LOH. Blood tests showed total testosterone 3.79 ng/mL (not ≤2.5 ng/mL) and free testosterone 7.5 pg/mL (at the threshold). At the patient’s request, a trial course of testosterone replacement therapy with testosterone enanthate was initiated, with tadalafil. Sexual function improved, and flushing and edema decreased after several days, followed by recurrence of edema, sweating, and fluctuating fatigue by three weeks. Symptoms persisted, and attribution remained unsettled. Psychiatric consultation was added.

This case visualizes how entry-point framing, biochemical borderline results, and symptom overlap can prevent diagnostic convergence. It supports a parallel-evaluation diagnostic design that prespecifies competing hypotheses and interprets treatment response as supportive but non-decisive evidence.

## Introduction

Late-onset hypogonadism (LOH) is a clinical concept that can affect sexual function, physical vitality, and psychological or behavioral domains [1-4]. In clinical practice, LOH functions as a framework that integrates symptoms with decreased testosterone [3,5]. It is not a disease entity confirmed by organ-specific findings in a single organ system [1,3]. Broader presenting complaints allow the same symptom to fit multiple pathophysiological mechanisms, which destabilizes diagnostic attribution. Mood, behavior, self-perception, and quality of life (QOL) show multidimensional associations with testosterone [1,6]. It is diagnostically important that psychological complaints can be framed as part of testosterone-related symptom complexes.

Psychological symptoms relevant to LOH, such as depressive symptoms, are nonspecific. When psychological distress becomes prominent, identical complaints can be attributed to endocrinological explanations or psychological frameworks [1]. Anxiety-related distress may present with prominent somatic complaints, and structured mental health assessment may be delayed in some care pathways. This report focuses on clinical situations where symptom overlap, including psychological distress, destabilizes diagnostic reasoning, rather than reviewing a specific disease label.

A further amplifier of instability arises from measurement practices and diagnostic thresholds. Endocrinological evaluation recommends fasting morning total testosterone (TT) measured with accurate assays, with confirmation by repeat blood tests [5,7,8]. However, consensus on cutoffs for TT and free testosterone (FT) is not uniform within the LOH field [3,9,10]. In borderline cases, classification as low can vary across operational definitions. Thus, operational variation in biochemical assessment can hinder diagnostic convergence in addition to symptom nonspecificity.

Regarding treatment, interpretations of testosterone replacement therapy (TRT) effects on mood and vitality depend on target populations and evaluation designs [11,12]. Associations between testosterone and QOL are reported, yet clinical fluctuations in mood and vitality arise from multifactorial determinants [6,11]. Therefore, etiologic simplification based solely on treatment response remains theoretically unstable. This issue directly links to whether TRT can function as a substitute for diagnostic reasoning.

This case report aims to present a structure in which diagnostic attribution becomes unstable in undiagnosed presentations with LOH-like complaints. The structure emerges when symptom nonspecificity, variability in diagnostic implementation, including measurement conditions and thresholds, and limits of treatment response interpretation overlap. Accordingly, this report visualizes, along the clinical course, a diagnostic bottleneck where endocrinological evaluation intersects with symptom interpretation that includes psychological distress.

## Case presentation

A 52-year-old Japanese man presented for evaluation. Height was 173 cm, weight 68.2 kg, and BMI 22.8. Baseline temperament was described as realistic, and self-control was described as weak. His history included outpatient psychosomatic care for sleep disturbance. Alcohol use and tobacco smoking were absent. Reported allergies included cherries and peaches. He lived with his wife, aged 46 years, his son, aged 14 years, and his daughter, aged 11 years. His father (82 years), mother (79 years), brother (54 years), and sister (49 years) were alive. He had been married for 21 years and had no history of divorce. Family history included esophageal cancer in his mother. Family history also included a developmental disorder in his daughter. Other relatives had no reported psychiatric disorders, dementia, or endocrine disorders. He lived separately from his parents, and his mother provided care for his father. He had been employed for 25 years and had held a managerial position for more than 10 years. In January 2025, he took a one-month leave of absence due to symptom exacerbation and later returned to work.

In November 2024, he visited Gastroenterology for abdominal pain, abdominal bloating, constipation, flushing, and insomnia. The workup resulted in constipation-predominant irritable bowel syndrome. Medications included a *Clostridium butyricum *probiotic, macrogol 4000 with electrolytes, linaclotide, and yokukansan (TJ-54, a Kampo medicine). Symptoms remained stable under this regimen.

In December 2024, a local clinic referred him to a rheumatology service for suspected rheumatoid arthritis. He had low-grade fever, arthralgia, and swelling of fingers and toes. Oral ulcers and lower-leg edema were absent. Rheumatoid factor (RF), anti-cyclic citrullinated peptide antibody (anti-CCP Ab), and antinuclear antibody (ANA) were negative, and C-reactive protein (CRP) showed no elevation. Ultrasonography showed no synovitis and no enthesitis. Symptoms improved with symptomatic treatment using oral loxoprofen alone. A transient viral infection was suspected, and follow-up ended.

In April 2025, stiffness and neuralgia recurred, and erectile dysfunction was also reported. Rheumatology reassessed the recurrence. Non-contrast CT from the neck to the pelvis showed no findings suggestive of collagen vascular disease. Non-contrast cervical spine MRI also showed no suggestive findings. In May 2025, palpitations, flushing, and dizziness worsened. Vital signs were stable, with oxygen saturation (SpO₂) of 98%, respiratory rate (RR) of 14/min, and heart rate (HR) of 78/min. The temperature was 36.5℃, and the BP was 105/78 mmHg. ECG showed sinus rhythm at 63/min with PR 0.178 seconds, QRS 0.095 seconds, and QTc 0.395 seconds. ST-T change and conduction disturbance were absent. Blood tests assessed thyroid disease, cardiac disease, autoimmune disease, and coagulation abnormalities, with no abnormal findings in Table 1.

**Table 1:** Laboratory investigations across time points Serial laboratory results during follow-up in 2025. Values are reported by test date. A dash (—) indicates not measured. WBC, white blood cell count; Hb, hemoglobin; Hct, hematocrit; PLT, platelet count; CRP, C-reactive protein; BUN, blood urea nitrogen; Cr, creatinine; eGFR, estimated glomerular filtration rate; AST, aspartate aminotransferase; ALT, alanine aminotransferase; tBil, total bilirubin; TP, total protein; Alb, albumin; Na, sodium; K, potassium; Cl, chloride; Ca, calcium; Glu, glucose; HbA1c, hemoglobin A1c; TSH, thyroid-stimulating hormone; fT3, free triiodothyronine; fT4, free thyroxine; cTnT, cardiac troponin T; NT-proBNP, N-terminal pro-B-type natriuretic peptide; RF, rheumatoid factor; anti-CCP Ab, anti-cyclic citrullinated peptide antibody; ANA, antinuclear antibody; MMP-3, matrix metalloproteinase-3; PR3-ANCA, proteinase 3 anti-neutrophil cytoplasmic antibody; MPO-ANCA, myeloperoxidase anti-neutrophil cytoplasmic antibody; anti-dsDNA (RIA), anti–double-stranded DNA antibody (radioimmunoassay); anti-SS-A/Ro Ab, anti–Sjögren’s-syndrome-related antigen A/Ro antibody; anti-U1 RNP Ab, anti-U1 ribonucleoprotein antibody; u-Pro, urine protein; u-RBC, urine red blood cells; u-WBC, urine white blood cells; HPF, high-power field; PSA, prostate-specific antigen; LH, luteinizing hormone; FSH, follicle-stimulating hormone; PRL, prolactin; TT, total testosterone; FT, free testosterone.

Test	Unit	1/22	5/12	6/30	12/11
WBC	/µL	4900	5200	5100	5400
Hb	g/dL	13.8	14.2	13.7	14.6
Hct	%	44.2	42.9	42.4	45
PLT	/µL	24.9	23.5	25.2	26.9
CRP	mg/dL	0.1	0.15	0.04	—
BUN	mg/dL	10	11	12	10
Cr	mg/dL	0.73	0.79	0.85	0.77
eGFR	mL/min/1.73m^2^	88.6	81.2	75	83.1
AST	U/L	14	16	14	14
ALT	U/L	21	22	15	23
tBil	mg/dL	0.75	0.52	0.69	0.48
TP	g/dL	7	—	6.7	6.6
Alb	g/dL	4.4	4.3	4.3	4.1
Na	mmol/L	143	142	144	140
K	mmol/L	4.2	4.2	3.9	4.2
Cl	mmol/L	105	105	106	104
Ca	mg/dL	—	—	9.4	9.2
Glu	mg/dL	107	110	108	103
HbA1c	%	5.8	5.8	—	5.9
TSH	µIU/mL	1.63	1.08	—	0.89
fT3	pg/mL	3.7	3.6	—	3.3
fT4	ng/dL	1.2	1.3	—	1.1
cTnT	pg/mL	—	0.005	—	—
NT-proBNP	pg/mL	28.2	—	—	—
RF	IU/mL	<5.0	<5.0	—	—
anti-CCP Ab	U/mL	<0.6	—	—	—
ANA	—	1:40	—	—	—
MMP-3	ng/mL	—	76.2	—	—
PR3-ANCA	U/mL	<1.0	—	—	—
MPO-ANCA	U/mL	<1.0	—	—	—
anti-dsDNA(RIA)	IU/mL	<2.0	—	—	—
anti-SS-A/Ro Ab	U/mL	( - )	—	—	—
anti-U1 RNP Ab	U/mL	( - )	—	—	—
u-Pro	mg/day	11.5	—	—	—
u-RBC	/HPF	<1	<1	1-4	—
u-WBC	/HPF	1-4	<1	<1	—
PSA	ng/mL	—	—	0.689	—
LH	mIU/mL	—	—	10.6	—
FSH	mIU/mL	—	—	11.2	—
PRL	ng/mL	—	—	16.2	—
TT	ng/mL	—	—	3.79	—
FT	pg/mL	—	—	7.5	—

At the rheumatology assessment, erectile dysfunction co-occurred with palpitations, flushing, and dizziness. Organic disease appeared unlikely. Sexual dysfunction co-occurred with unexplained somatic symptoms. Internal medicine prioritized suspected LOH and referred him to urology. Mental health assessment at that point was limited to interview items. He denied fatigue and difficulty initiating sleep, and work functioning was preserved. Comprehensive symptom quantification and standardized rating scales were not used.

In June 2025, he presented to urology. He reported fatigue as the dominant symptom, with awakening after sleep onset, flushing, palpitations, erectile dysfunction, stiffness, and edema. Due to outpatient scheduling, a single blood draw was performed at 16:00. TT was 3.79 ng/mL and did not meet the Japanese diagnostic criterion of 2.5 ng/mL or less [9]. FT was 7.5 pg/mL and matched the Japanese diagnostic threshold [9]. Luteinizing hormone (LH) was 10.6 mIU/mL, and follicle-stimulating hormone (FSH) was 11.2 mIU/mL, both mildly elevated. Prolactin (PRL) was 16.2 ng/mL and was mildly elevated, and pituitary-related symptoms were absent. Pathological findings were limited, and secondary causes were less strongly suggested. Urinalysis showed no abnormalities. Interview screening suggested low risk for sleep apnea symptoms. Edema was not prominent, and weight change was absent. Creatinine (Cr) and estimated glomerular filtration rate (eGFR), albumin (Alb), aspartate aminotransferase (AST), alanine aminotransferase (ALT), and urine protein showed no abnormalities (Table 1). Hemoglobin (Hb) was 13.7 g/dL, hematocrit (Hct) was 42.4 percent, and prostate-specific antigen (PSA) was 0.689 ng/mL.

For suspected LOH, the patient requested TRT. Risks were explained, including fertility-related risks, before initiation. Testosterone enanthate 250 mg IM was administered as a trial course for three injections. Tadalafil 5 mg was also initiated at the patient’s request. Sexual function improved after initiation. From three days after TRT, flushing decreased, and edema of the hands and feet decreased, with resumption of exercise. From three weeks after TRT, recurrence of edema, sweating, and fatigue was reported. Fatigue remained fluctuating and did not fully resolve after TRT initiation. Psychiatry consultation followed based on persistent symptoms. After TRT discontinuation, sexual function remained maintained with tadalafil.

In December 2025, he presented to psychiatry. His chief complaint was daily persistent flushing, palpitations, and restlessness. Additional complaints included light sleep, anxiety, scattered thoughts, fatigue, and forgetfulness. Symptom severity fluctuated, and worsening occurred upon awakening. Sleep was light, without awakening after sleep onset, and total sleep duration was six hours. Appetite was preserved. A sense of emptiness was reported, while guilt, worthlessness, and suicidal ideation were denied. No paroxysmal episodes occurred during the preceding two months. Grooming and social decorum were preserved during the interview. Body habitus was slender, and facial expression appeared calm. Cooperation was good, and speech quantity was relatively increased. Social and occupational functioning were preserved. Regarding the Diagnostic and Statistical Manual of Mental Disorders - 5 (DSM-5) major depressive episode, two of the nine criteria persisted: depressed mood and diminished interest or pleasure [13].

Diagnostic criteria for depressive disorder were not met. Findings suggesting substance-induced or medication-induced etiology were limited. A history of manic episodes was absent. For DSM-5 panic disorder criteria, recurrent unexpected panic attacks were considered [13]. Symptoms included palpitations, sweating, tremor, dizziness, fear of losing control or sanity, and fear of dying. Anticipatory anxiety persisted for more than one month. Clear attack symptoms during the preceding two months were not recognized by the patient. Prior recurrent attack-like symptoms also informed the assessment. Management proceeded as suspected panic disorder with follow-up observation. Alprazolam 0.4 mg was prescribed as needed for attacks. Lemborexant 2.5 mg was prescribed as needed for insomnia.

In February 2026, he visited both urology and psychiatry. In urology, sexual function was maintained with tadalafil, and continuation of tadalafil was recommended. In psychiatry, symptoms were described as stable within self-control under as-needed alprazolam and lemborexant. No medication changes were made, and follow-up was continued.

## Discussion

This case showed a course in which overlapping symptoms were interpreted in accordance with each specialty’s clinical assessment, leading to fluctuating diagnostic framing. The key point is not an early binary decision between LOH and a psychiatric disorder. In undiagnosed inflow cases, the perspective of the first department visited often becomes the starting point for subsequent reasoning [14-16]. Symptom clusters as chief complaints then tend to converge early toward a specific disease label. As a result, the entry-point framing becomes fixed, and competing hypotheses received insufficient consideration later. Accordingly, this report’s contribution to diagnostic reasoning lies in discussing a procedure that avoided premature disease labeling at an early stage and evaluated multiple hypotheses in parallel. It also discusses re-evaluation based on the clinical course and test results and addresses a diagnostic design that clarified the diagnostic role of treatment response. 

This case also suggests a system-level influence on diagnostic framing. The care pathway prioritized organic and physical evaluation in the early phase. Mental health assessment remained brief at the referral point. In men with overlapping LOH-like and anxiety or panic-like complaints, integrated or parallel evaluation may reduce delayed recognition of psychiatric conditions.

LOH diagnosis is generally defined as a combination of symptoms and consistently low testosterone [9]. However, diagnostic uncertainty is noted, reflecting symptom nonspecificity and frequent discordance between symptoms and low testosterone [1,3]. Therefore, foregrounding the disease label (LOH) at the outset in undiagnosed inflow cases can readily delay competing hypotheses. The phenotype label proposed in this report differs from a rating scale and constitutes a classification that forms the backbone of diagnostic reasoning [17]. In other words, a scale serves as a measurement tool, whereas a phenotype label serves as a clinical tag. The tag determines which hypotheses run in parallel from the beginning, what to verify next, and where to update hypotheses.

Candidate phenotype labels in this case are as follows: Candidate A is an autonomic-dominant, fluctuating phenotype in which palpitations, flushing, and dizziness recurred as chief complaints. Candidate B is a phenotype with concomitant sexual dysfunction characterized by erectile dysfunction. Importantly, these labels are not diagnostic names. For example, assigning Candidate A readily evokes a parallel-evaluation design that advances LOH evaluation while avoiding delay of anxiety and panic hypotheses. This approach provides an entry-point framing that beginners can reproduce.

In this case, vital signs, ECG, blood tests, CT, and MRI were obtained during symptom exacerbation to prioritize evaluation for organic disease. Negative findings accumulated for collagen vascular disease, thyroid dysfunction, and cardiac disease, yet symptoms persisted, and psychiatric hypotheses later became predominant. The diagnostic point does not lie in exhaustive enumeration of exclusion items. Urgent-condition exclusion provides a prerequisite for subsequent parallel evaluation in undiagnosed inflow cases.

In this case, a single 16:00 blood draw showed FT in a low range, whereas TT did not fall below a typical cutoff. Clinical guidelines commonly prioritize repeatedly measured testosterone values for diagnosis, usually obtained in the early morning [5]. Accordingly, this report did not conclude LOH presence or absence from testosterone indices. Instead, it specifies the limits of the measurement design, and returning diagnostic reasoning to a parallel-evaluation framework is appropriate. Moreover, strict LOH criteria emphasize concomitant sexual symptoms, while details beyond erectile dysfunction are unavailable in this case [1]. Therefore, in similar undiagnosed inflow cases, it is useful to document what symptoms, including sexual complaints, emerge, to what degree, and over what time course. These data can be linked to the phenotype label Candidate B.

This case described a short-term response to TRT followed by subsequent recurrence. A common error is the use of a response to TRT as evidence for LOH or as grounds for excluding LOH. The LOH field combines symptom nonspecificity with uncertainty in diagnostic thresholds [3,4,18]. Response to TRT after a trial course is sometimes used as a differential diagnostic probe, yet inadequate evaluation readily entangles diagnostic reasoning [1,3]. Treatment-effect assessment, therefore, requires a prespecified evaluation design, including outcomes and assessment time points [19,20]. In this case, symptoms improved at three days after injection and recurred by three weeks after injection. Accordingly, several elements should be prespecified before initiating TRT.

First, the primary outcome should be fixed after domain-based decomposition [20]. Systematic domain-level documentation was not recorded. A single global outcome of better or worse does not support a correct diagnosis [20]. Repeated objective follow-up of domain-specific change using consistent instruments is useful [19,20].

Second, assessment time points should be fixed at two points: short-term and before the next administration. This case indicates that findings can differ after several days and after several weeks. Accordingly, the same domains should be recorded within the same framework at both time points.

Third, a definition of non-response to TRT should be prespecified and linked to hypothesis updating. Limited domain-level improvement should be treated not as LOH exclusion but as difficulty in explaining the presentation with a single hypothesis. This requires hypothesis reassessment and earlier initiation of parallel evaluation for competing hypotheses, including psychiatric disorders. It is clinically coherent to prespecify a transition rule that directs the next step according to evaluation results. Accordingly, TRT should not be used as a diagnostic test. TRT should be positioned as a therapeutic intervention, with prespecified outcomes and assessment time points. When the response to TRT is insufficient, the result should connect to hypothesis updating within the diagnostic reasoning process. This approach represents an implementable lesson derived from this case.

This report has several limitations. Testosterone measurement occurred once at 16:00 and did not meet repeated blood tests emphasized in diagnostic frameworks, usually in the morning [5,10]. Detailed sexual symptom information emphasized in strict LOH criteria remains unknown in this case, including sexual thoughts and reduced frequency of morning erections [1]. Domain-specific TRT outcomes were not recorded systematically. Responsiveness, therefore, cannot be linked quantitatively to diagnostic reasoning. For this reason, this report focuses on diagnostic design rather than diagnostic confirmation [19,20].

## Conclusions

This case shows overlap between LOH-like complaints and psychiatric disorder-like complaints, and diagnostic framing can shift with the care pathway. The contribution of this report is a diagnostic design that resists premature label convergence at entry, uses phenotype labels to keep competing hypotheses explicit, and moves from safety gating to parallel evaluation. In addition, the report makes explicit the limits of testosterone measurement design and clarifies that response to TRT is not diagnostic, linking insufficient response to hypothesis updating in similar undiagnosed inflow cases.
